# Parasystole in a Mahaim Accessory Pathway

**DOI:** 10.1016/s0972-6292(16)30780-x

**Published:** 2014-07-15

**Authors:** Chandramohan Ramasamy, Senthil Kumar, Raja J Selvaraj

**Affiliations:** Department of Cardiology, Jawaharlal Institute of Postgraduate Medical Education and Research, Puducherry, India

**Keywords:** Mahaim tachycardia, Parasystole, Automaticity

## Abstract

Automaticity has been described in Mahaim pathways, both spontaneously and during radiofrequency ablation. We describe an unusual case of automatic rhythm from a Mahaim pathway presenting as parasystole. The parasystolic beats were also found to initiate tachycardia, resulting in initial presentation with incessant tachycardia and tachycardia induced cardiomyopathy.

## Introduction

Mahaim pathways are atriofascicular accessory pathways with decremental, anterograde only conduction. The most common clinical manifestation related to these pathways is antidromic reentrant tachycardia. Less commonly, the pathway may be a bystander with atrioventricular nodal reentrant tachycardia or atrial tachycardia. Rarely, automaticity has been reported from the pathway, manifesting as ectopic beats during sinus rhythm or as an automatic tachycardia [1,2]. Parasystole is a condition where an ectopic focus is unaffected by the underlying rhythm due to entrance block. Parasystole has been reported from atrial musculature, ventricular musculature and His-Purkinje system, but not from Mahaim pathways.

## Case Report

**Figure 1:**
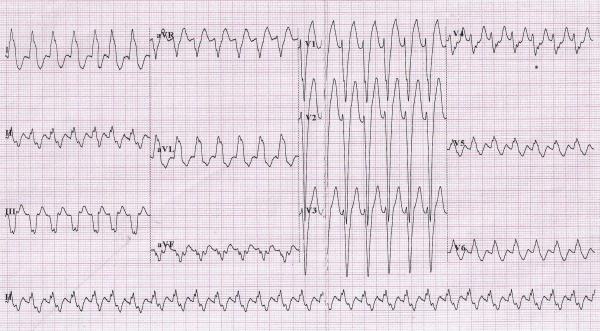
ECG at presentation. Regular, wide QRS tachycardia at a rate of 170 beats per minute with left bundle branch block morphology, left axis deviation and 1:1 VA association.

**Figure 2:**
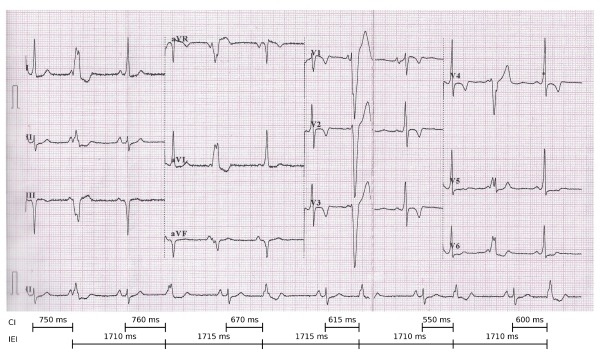
ECG immediately after termination with adenosine. Note that the rhythm strip is a continuous strip while the other leads show the same 2.5 second period. Preexcitation is seen in the sinus beats with negative delta wave in III, aVF, positive delta wave in V1 and abrupt transition in V2 indicating posteroseptal pathway. Ectopic beats are seen after each sinus beat with the same morphology as that during tachycardia. Intervals marked below show that there is significant variation in the coupling intervals from the sinus beats to the ectopic beats, while the interectopic intervals remain relatively constant. CI = Coupling Interval, IEI = Inter-ectopic interval.

**Figure 3:**
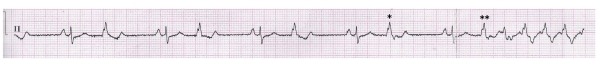
Reinitiation of tachycardia. Rhythm strip of lead II shows same rhythm as in figure 2. There is progressive subtle shortening of the coupling interval of the ectopics. The first three ectopic beats do not conduct to the atrium due to collision with sinus beats, while the fourth (*) conducts to the atrium and the fifth (**) conducts and initiates tachycardia.

A 32 year-old-male initially presented elsewhere with persistent palpitations and breathlessness. He was found to have tachycardia and severe left ventricular dysfunction. He was treated with DC cardioversion, started on Amiodarone and referred to us for further management. A 12-lead electrocardiogram (ECG) at admission showed wide QRS tachycardia at a rate of 200 beats per minute with left bundle branch block morphology, left axis deviation, precordial transition at V5 and 1:1 ventriculo-atrial association (Figure 1). Tachycardia terminated with 6 mg adenosine administered as an intravenous bolus. ECG taken in sinus rhythm showed preexcitation with an LBBB morphology, left axis and early precordial transition consistent with a posteroseptal accessory pathway. Wide QRS complexes, similar in morphology to the QRS complex during tachycardia, were noted interspersed with the sinus beats. These broad complex beats were not preceded by P waves and were not constantly coupled to the preceding sinus beat, while the interval between these beats was constant (Figure 2). Re-initiation of tachycardia was noted to occur following some of these beats (Figure 3).

During electrophysiology study, presence of a right sided atriofascicular pathway with decremental, anterograde only conduction (Mahaim pathway) and a posteroseptal accessory pathway with antegrade only conduction and long effective refractory period were confirmed. Reentrant tachycardia with the Mahaim pathway as the anterograde limb and the atrioventricular node as the retrograde limb was easily induced with ventricular pacing. The Mahaim pathway was successfully ablated. No tachycardia was inducible after ablation. In view of this and the low risk conduction properties, the posteroseptal accessory pathway was not ablated.

## Discussion

Mahaim pathways are atriofascicular pathways with atrioventricular node like properties. Automaticity of the pathway is typically seen during radiofrequency ablation [1], but also sometimes spontaneously [2]. Parasystole represents an independent ectopic rhythm that competes with sinus rhythm. It appears on the ECG as unifocal ectopic beats with a variable coupling interval, while the interectopic intervals are the same or multiples of a common cycle length. Due to the entrance block, this focus is not depressed or overdriven by the normal ventricular conduction.

In this patient, parasystole was seen arising from the Mahaim pathway. Parasystole can rarely appear like bigemini due to modulation resulting in near-synchronization with the sinus beats [3]. In this patient, there were small variations in the coupling intervals of the ectopic beats while the interectopic interval was nearly constant, proving it was parasystole. Interestingly, the parasystolic beats were found to initiate tachycardia when the coupling interval was short enough to result in ventriculo-atrial conduction. We presume that this resulted in repeated induction of tachycardia in this patient leading to the initial presentation with tachycardia induced cardiomyopathy. Other interesting aspect of the case was the presence of an additional atrioventricular accessory pathway, which is an uncommon, but well reported association [4]. This posteroseptal pathway, however, did not require to be ablated because it was not contributing to any tachycardia and had low risk antegrade conduction properties.

## Conclusion

This is an unusual case of parasystolic rhythm from a Mahaim pathway with the parasystolic beats initiating reentrant tachycardia. This was probably responsible for the unusual initial presentation with incessant tachycardia and tachycardia induced cardiomyopathy.
